# Cyclodextrin-Containing Hydrogels: A Review of Preparation Method, Drug Delivery, and Degradation Behavior

**DOI:** 10.3390/ijms222413516

**Published:** 2021-12-16

**Authors:** Jiayue Liu, Bingren Tian, Yumei Liu, Jian-Bo Wan

**Affiliations:** 1 State Key Laboratory of Quality Research in Chinese Medicine, Institute of Chinese Medical Sciences, University of Macau, Macao 999078, China; liujiayue0331@163.com; 2 School of Chemical Engineering and Technology, Xinjiang University, Urumqi 830046, China; tianbingren1@163.com

**Keywords:** cyclodextrin, hydrogel, preparation, release, degradation

## Abstract

Hydrogels possess porous structures, which are widely applied in the field of materials and biomedicine. As a natural oligosaccharide, cyclodextrin (CD) has shown remarkable application prospects in the synthesis and utilization of hydrogels. CD can be incorporated into hydrogels to form chemically or physically cross-linked networks. Furthermore, the unique cavity structure of CD makes it an ideal vehicle for the delivery of active ingredients into target tissues. This review describes useful methods to prepare CD-containing hydrogels. In addition, the potential biomedical applications of CD-containing hydrogels are reviewed. The release and degradation process of CD-containing hydrogels under different conditions are discussed. Finally, the current challenges and future research directions on CD-containing hydrogels are presented.

## 1. Introduction

Many studies in the material science field have explored diverse materials that could improve drug delivery, thus ensuring effective treatment of various diseases [1,2]. In the past few decades, numerous materials have been synthesized and applied in drug delivery systems [3,4]. Particularly, several clinical trials have explored the development of hydrogels and their potential applications [5]. The physical properties of hydrogels are biocompatible to organisms. Therefore, they have become indispensable materials in several biomedical applications [6]. Hydrophilic groups present in the hydrogel network enable the hydrogel to interact with water molecules in the surrounding environment. As a result, the hydrogel can absorb large amounts of water to maintain its structure and viscoelasticity. The volume changes of hydrogels can be controlled by changing parameters such as composite molecules and cross-linking density in different environments. Hydrogels are mainly synthesized in the water phase, therefore the introduction of cross-linking points throughout the hydrogel network is important in preventing the hydrogel from dissolving. According to the different cross-linking properties in the hydrogel network, hydrogels are mainly divided into two categories, including physical and chemical cross-linked hydrogels [7,8]. Physically cross-linked hydrogels are formed through physical interactions between the polymers that form hydrogels. On the other hand, chemically cross-linked hydrogels are formed by covalent bonds.

These properties enable the use of hydrogels as macromolecular platforms in drug delivery, wound healing dressings, and implant coatings [9,10,11]. Previous studies developed controllable, localized drug release systems for hydrogel systems [12,13]. Commonly used methods for drug loading include forming unstable chemical bonds to covalently bind drug molecules to the hydrogel matrix or employing non-covalent methods to encapsulate drug molecules into the hydrogel. Although the hydrogel can effectively control the release time when the drug is loaded through a covalent method, the synthesis process and the loading amount of the drug are not satisfactory [14,15]. On the contrary, physically trapping drug molecules into the hydrogel is simpler, and results in a hydrogel with a higher drug loading capacity. Drugs are mainly loaded to hydrogels through hydrophobic, electrostatic, or hydrogen bond interactions. However, most hydrogels are hydrophilic in nature, therefore, loading some hydrophobic drugs is not effective. Bringing hydrophobic regions into the hydrogel network can increase drug loading capacity and reduce the burst effect of the drug at the initial stage of entering the body [16,17,18].

Cyclodextrin (CD) is a cyclic oligosaccharide consisting of glucopyranoside units linked through α-1,4 glycosidic bonds obtained [19]. Structural analysis shows that CD is characterized by external hydrophilicity and internal hydrophobicity. Due to its unique cavity structure, hydrophobic molecules can be loaded to form an inclusion complex in dynamic equilibrium [19]. Because of several hydroxyl groups existing in the external structure, CD could form physically cross-linked hydrogel through intermolecular forces. In addition, CD can be connected to form a chemically cross-linked hydrogel network through covalent bonds. Several studies have explored these two different types of CD-containing hydrogels [2,20]. 

Although CD-containing hydrogels are still in the basic research stage, they have prominent advantages in different applications [21,22,23,24,25,26] and remarkable potential in biomedical applications, thus improving human health (Table 1). This review is aimed to discuss and summarize the development of CD-containing hydrogels in drug delivery. The traits of hydrogels are classified and discussed based on the different preparation methods. Furthermore, the potential applications of these hydrogels are summarized. In addition, the release rate of drugs and degradation of hydrogels are explored. Further, we summarize prospects for the future development of CD-containing hydrogels based on previous research findings.

## 2. Preparation Methods of CD-Containing Hydrogels

**Figure 1:**
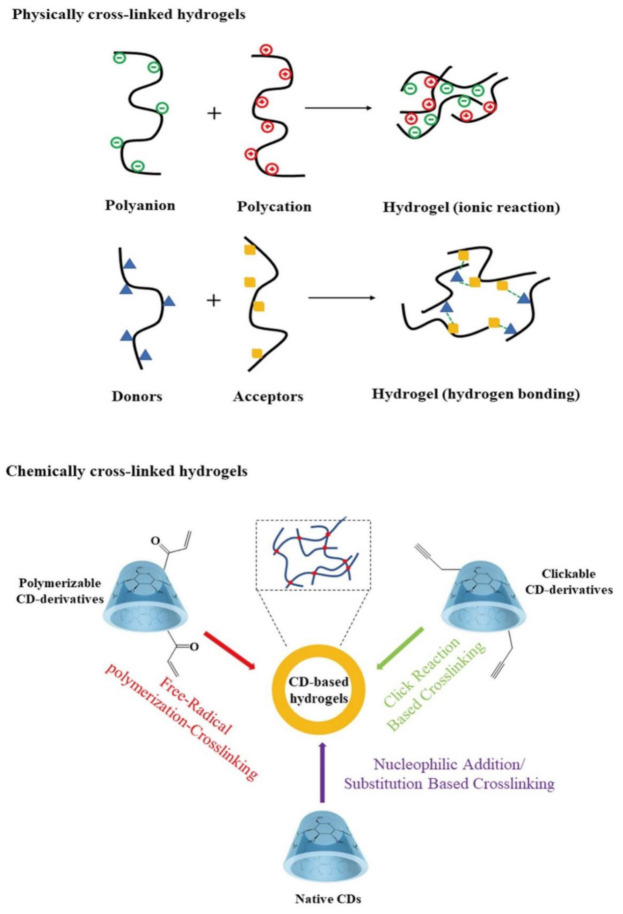
Schematic representation of common methodologies for preparation of CD-containing hydrogels.

Studies on the use of the unique cavity of CD to encapsulate and deliver drugs are ongoing [40,41,42]. CD hydrogels can be divided into two groups including physically crosslinked hydrogels and chemically cross-linked hydrogels (Figure 1).

Table 2 summarizes some examples of the preparation methods of physically cross-linked or chemically cross-linked cyclodextrin-containing hydrogels. Although physical hydrogels are non-toxic, they still have some shortcomings, such as low mechanical strength, and their pore size cannot be easily adjusted [43]. Physically cross-linked hydrogels are stable enough to prevent them from dissolving in water [44,45]. The methods involved in chemical cross-linking include free radical polymerization cross-linking-based methods; nucleophilic addition/substitution-based methods; cross-linking methods based on ‘click’ reactions and incorporation of CDs through post-gelation attachment [2]. The chemical activity produces permanent hydrogels through the covalent interaction of polymer and crosslinker functional groups. Polymerization into a hydrogel network produces fine-tuned hydrogels through chain growth, addition, or condensation reactions [46,47]. 

In addition, hydroxyl groups occur at different positions in the CD molecule, thus they have different effects on the formed derivatives [68]. CD formed from glucopyranose has two types of hydroxyl groups, one is the primary hydroxyl group at the 6- position and the other is the secondary hydroxyl group at the 2- and 3- positions [69]. On the other hand, the primary hydroxyl group outside the cyclodextrin is free to move. In addition, the acidic and basic properties of the three hydroxyl groups are significantly different. The hydroxyl group at the 6- position is basic; the hydroxyl group at the 2- position is acidic (pKa = 12.1), whereas the 3- hydroxyl group is not easily modified. Therefore, the chemical modification process of CD is affected by the nucleophilicity of the hydroxyl group and the modification reagent. Under normal reaction conditions, the 6-position hydroxyl group is the most active and easily participates in the reaction after attack by electrophiles. Electrophiles can also attack other positions, including the less popular hydroxyl groups (2- and 3-). More than 40% of the compounds have low water-solubility properties. In addition, some compounds have poor stability or poor taste [70,71,72]. CD has become the ideal material to solve solubility, light stability, and poor taste limitations. Different CDs have different sizes of hydrophobic cavities, so it is important to choose specific CD derivatives based on the molecular size of the target molecule [73]. Owing to the low solubility of parent CD, its further application in medicine was limited. The preparation of water-soluble derivatives is important for improving the application of CD in drug delivery systems. Therefore, different CD-based derivatives have been developed to improve water solubility and functionality of natural CD.

## 3. The Promising Application of CD-Containing Hydrogels for Drug Delivery

In this section, we classify and discuss different types of CD-containing hydrogels based on the preparation method (Table 3). The release behavior of drugs after the introduction of different CD into hydrogels will be explored. Besides, the potential application of the prepared physical/chemical CD-containing hydrogel in the medical field will be discussed.

### 3.1. Physically Cross-Linked CD-Containing Hydrogels

The physically cross-linked hydrogel formed by intermolecular interaction does not cause damage to the environment during the preparation process [49,80]. Therefore, physically cross-linked hydrogels have wide applications in the medical field. This section mainly summarizes and analyzes potential applications of physically cross-linked CD-containing hydrogels loaded with different drugs in different medical fields (Table 4). Because in the process of preparation, the physically cross-linked CD-containing hydrogels don’t involve the chemical cross-linking agents and chemical solvents, so physical gelatinization and drug encapsulation simultaneously have important research and application value, especially in the aspect of oral administration medicine, ocular delivery system, wound dressing materials, local drug delivery system to treat cancer, etc.

Sajeesh et al. used chitosan, methacrylic acid, and polyethylene glycol to prepare hydrogels, and then added insulin-loaded methyl-β-CD into the hydrogels [86]. Final hydrogel particles were formed through the interaction between CD and the hydrogel matrix. The encapsulation efficiency of the inclusion compound and insulin encapsulated by hydrogel microparticles were evaluated. No significant differences were observed in the encapsulation efficiency of the two systems. Insulin concentrations of 0.5 and 1 mg/mL showed an encapsulation efficiency of the inclusion compound preparation of 87% and 82%, respectively. The efficiency of the non-encapsulated system was 90% and 85% for insulin concentration at 0.5 and 1 mg/mL, respectively. In addition, in vitro drug release experiment showed that the inclusion compound encapsulated by the hydrogel exhibited pH responsiveness. At pH = 1.2 and 7.4, the amount of insulin released under more acidic conditions was significantly lower compared with that under neutral conditions over the same period. The effect of oral delivery of insulin by CD hydrogel inclusion compound microparticles was studied using streptozotocin-induced diabetic rats. The experimental results exhibited that insulin loading, and release characteristics of the hydrogel matrix were not affected by the complexation of CD. In addition, CD compound insulin coated by hydrogel particles effectively reduced blood sugar levels in diabetic animals.

To deliver oral hypoglycemic drugs into the body, Okubo et al. employed the host-guest interaction between hydrophobically modified hydroxypropyl methylcellulose and CD to develop and prepare heat-responsive injectable drug sustained-release hydrogel. The hydrogel was prepared by mixing the CD inclusion compound containing insulin with cellulose. Due to the interaction between the stearyl group of cellulose and the β-CD cavity, the hydrogel underwent a thermal gelation reaction near the human body temperature. The newly prepared hydrogel was effective for up to 24 h after subcutaneous administration of mice. Notably, pharmacokinetic experiment results showed that the hydrogel released insulin which reduced the blood sugar levels [87].

Drugs can be delivered into the human intestinal tract through oral administration to achieve high efficacy [88]. The solubility of berberine hydrochloride could be enhanced by β-CD (25 °C, 11.41 mM). CD-loaded berberine hydrochloride-containing bacterial cellulose hydrogel was prepared by physical adsorption, and its drug loading (34%) was higher compared with that of hydrogels prepared without CD (17.2%). In vitro, drug release experiments revealed that the hydrogel achieved sustained drug release (t > 70 h) under different pH conditions (pH = 1.2, 6.8, and 12.1) of the gastrointestinal fluid. In vitro antibacterial experiments exhibited that hydrogels had better antibacterial effects (*E. coli, S. aureus,* and *P. aeruginosa*), thus laying the foundation for oral drug delivery [54].

To speed up the healing of wounds on the skin surface and prevent bacterial infections, some wound dressings are prepared through the physical synthesis method [89,90]. Sodium alginate and chitosan are mixed to form a hydrogel matrix, and then β-CD inclusion compound containing curcumin is added as an active ingredient. It is reported that sodium alginate and chitosan adsorb each other through electrostatic interaction. The mechanical properties of hydrogel materials are significantly improved by an increase in calcium chloride content. In the active ingredient release study, the hydrogel displayed a sustained release effect (t > 48 h), which was not affected by the addition of calcium chloride. The curcumin-loaded hydrogel showed a good inhibitory effect on the growth of *E. coli* (73.95%) and *S. aureus* (71.59%). Toxicity studies displayed that the hydrogel was non-toxic to NCTC clone 929 cells and normal human dermal fibroblasts [48]. A similar study used hydroxypropyl-β-CD as a drug carrier for loading curcumin and added silver nanoparticles with antibacterial activity to the hydrogels. These hydrogels showed broad-spectrum antibacterial activity and antioxidant properties and can be used for wound dressing. In addition, the hydrogels showed good cell compatibility with different cell lines, including Panc 1 (human pancreatic ductal adenocarcinoma), U251 (Human brain glioma U251 cell line), and MSTO (human mesothelioma). Its high moisture content and good transparency further promote its application potential for the treatment of chronic wounds [82].

Because most people do not pay much attention to hygiene, the risk of pathogenic microorganisms coming into contact with the body is high. The eye, an organ that is directly in contact with the external environment, often suffers from keratitis due to infection by pathogens [91]. If keratitis is left untreated, it may cause permanent vision damage. Eye drops are the preferred method of drug delivery for the timely treatment of infections [92]. However, when eyes are stimulated by the outside environment, the number of blinks and secretion of tears will increase, thus most of the eye drops do not reach the infected areas. Therefore, the frequency of administration of eye drops is increased to increase efficacy. Hewitt et al. explored the possibility of adding drugs to contact lenses. They selected pig eyes as the research object and prepared a hydrogel with β-CD loaded with chlorhexidine. Antibacterial analysis showed that the drug-containing contact lenses delivered a high amount of chlorhexidine to the cornea within 24 h. Although the contact lens loaded with chlorhexidine β-CD failed to improve the drug delivery effect, it was able to deliver the drug to the cornea. In addition, β-CD hindered drug release in the hydrogel matrix. Continuous irrigation with simulated tear fluid can significantly reduce the amount of drug delivered to the cornea. Chlorhexidine retains antibacterial activity in all methods of administration. The hydrogel contact lens injected with chlorhexidine showed a significantly higher effect on the cornea, whether it was used multiple times or once compared with eye drops. Therefore, this method can be used to reduce the number of administrations thus improving patient tolerance degree [81].

### 3.2. Chemically Cross-Linked CD-Containing Hydrogels

The chemically cross-linked CD-containing hydrogels are not easily degraded in the external environment, thus reducing the number of hydrogels used during the application, ultimately mitigating side effects by the hydrogel drug delivery system (Table 5). Chemical methods can be used to prepare chemically cross-linked CD-containing hydrogels which possess much higher stability than the physically cross-linked CD-containing hydrogels, comparatively speaking. Therefore, the application scope of chemically cross-linked CD-containing hydrogels has been extended to some extent by changing the properties of hydrogels through different chemical reactions., especially in the aspect of injectable nanocarriers, cancer therapy, transdermal drug delivery, tissue engineering, regenerative medicine, wound healing, oral drug delivery, etc.

Several studies are currently exploring controlled drug delivery systems based on CD-containing hydrogels [103,104,105,106]. Xia et al. used new hesperidin-copper (II) (NH-Cu (II)) as the model drug, then added it into a hydrogel composed of carboxymethyl cellulose (CMC), cellulose nanocrystals (CNC), and hydroxypropyl-β-CD. Citric acid was used as a cross-linking agent to prepare a natural hydrogel film which exhibited controllable swelling behavior. The different dynamic behaviors of NH-Cu (II) in the hydrogel film were then explored. Drug release studies showed that the hydrogel had a sustained release effect at different temperatures. Furthermore, it had different swelling behavior under different pH and different ion concentrations. The swelling kinetics followed the Fick diffusion and Schott second-order kinetic model. In addition, the addition of CNC into hydrogel film changed the mechanical properties, thermal stability at high temperatures, swelling rate, salt sensitivity, and pH sensitivity of the hydrogel film in different solutions. Moreover, CNC greatly improved the loading and encapsulation efficiency of the hydrogel film. The addition of 4% CNC showed an optimal loading efficiency of 753.75 mg/g and a cumulative release rate of 85.08%. The hydrogel membrane showed good cell compatibility and was non-cytotoxic, thus it can be used as a potential drug delivery and controlled release system for wound dressing [63].

Targeted delivery of anti-cancer drugs is one of the most effective treatment methods for tumors [107]. Therefore, direct injection of the drug-containing hydrogel at the tumor area to maximize drug concentration improves the efficacy of the drug. Hyaluronic acid derivative and functionalized CD can be linked by a covalent bond to prepare an injectable hydrogel, and doxorubicin is successfully loaded in the CD cavity. In vitro release experiments of the drug-loaded hydrogels displayed a sustained-release effect. In this case, the hydrophobic interaction between doxorubicin and CD cavity resulted in drug retention in the hydrogel, thereby slowing its diffusion through the three-dimensional network. Notably, after 32 days of incubation, only 50% of the drug load was released in the medium without a significant burst effect. In addition, the hydrogel reduced the size of solid tumors in mice. Moreover, histological analysis of the heart of treated mice displayed showed no cardiotoxicity after treatment of the tumor with the drug-loaded hydrogel. These results implied that this drug-loaded hydrogel is an effective biomedical device to locally treat unresectable solid tumors or for preventing regeneration of residual tumors and inhibiting disease recurrence [99]. 

Subcutaneous administration of drugs has some side effects. For example, when treating diabetes, people prefer injecting insulin under the skin. However, daily insulin injection is associated with adverse effects, such as hypoglycemia, allergies, and peripheral hyperinsulinemia [102]. Therefore, an oral insulin delivery system can be used to avoid these side effects. A previous study reports oral hydrogen comprising CD and chitosan as raw materials and a water-soluble carbodiimide as cross-linking agent. SEM (scanning electron microscope), FTIR (Fourier transform infrared spectroscopy), XRD (X-Ray diffraction), and swelling experiments indicated the hydrogel had a porous structure. Insulin release behavior was shown to be triggered by in vitro pH. Notably, insulin was successfully retained in the stomach environment and slowly released after passing through the intestine. The stability of insulin secondary structure was studied by circular dichroism and fluorescence spectrophotometry. Analysis experiment revealed no significant difference in secondary structure between native insulin and released insulin. Furthermore, the hydrogel particles exhibit non-cytotoxicity and were mainly transported in the Caco-2 cell monolayer through paracellular pathways. Different insulin-loaded hydrogel microparticles were applied to diabetic mice to evaluate the effectiveness of hydrogel sustained-release microparticles in delivering insulin in the body. Insulin-loaded hydrogel particles significantly and continuously (6–12 h) reduced blood sugar levels in diabetic mice compared with subcutaneous injection. In summary, these findings indicate that hydrogel is a promising oral drug carrier to achieve sustained release [102].

Previous studies explored the use of chemically cross-linked hydrogels and compared them with physically cross-linked hydrogels for the treatment of eye diseases [108]. Cyclosporine is a commonly used drug for the treatment of various immune-mediated ocular surface diseases [109]. However, the poor water solubility of cyclosporine limits its application. Only two topical formulations of cyclosporine have been approved for the treatment of dry eye syndrome, Resis (Allergan, USA). An anionic emulsion (0.5 mg/mL cyclosporine) was approved by the FDA in 2002, and Ikervis was recently approved in Europe (Santen, Tampere, Finland), a cationic nanoemulsion containing 1 mg/mL cyclosporine [110,111,112]. Sodium hyaluronate and hydroxypropyl-β-CD are used as raw materials to prepare a chemically cross-linked hydrogel. Cyclosporin is loaded into the hydrogel by dipping to avoid degradation of cyclosporin during the cross-linking reaction. Interestingly, changing the weight ratio of the raw materials in the hydrogel adjusts the swelling and the rate of drug release. The swelling plays a key role in the feasibility of the drug penetrating through the sclera and accumulating into the eye tissue. In vitro drug release, experiments show that the hydrogel has a release time of up to 8 h. Therefore, chemically cross-linked hydrogels are effective systems for drug release to the ocular surface, however, the effectiveness should be confirmed in preclinical studies [67]. 

The polymerization system of the hydrogel obtained by CD polymerization and swelling has very few pores. Khalid et al. used β-CD, acrylic acid, and acrylamide to prepare β-CD nanosponges for loading dexibuprofen. Water solubility experiments showed that the water solubility of dexibuprofen was increased by 6.3 times after loading it to the hydrogel. The particle size of this material was 275.1 ± 28.5 nm. The swelling index of the nanosponge under weakly acidic conditions (pH = 6.8) was 3, and up to 89% dexibuprofen was successfully released within 30 min under this condition. An acute oral toxicity study using rats showed no toxicity-related conditions, death, adverse clinical symptoms and had no toxicological changes in hematology, clinical biochemistry, and histology. Therefore, the β-CD nanosponges prepared through the optimized condensation method may be superior compared with other β-CD nanoformulations, thus they improve oral administration of lipophilic drugs such as dexibuprofen [97].

## 4. Simulated Degradation Behavior for CD-Containing Hydrogels

**Figure 2:**
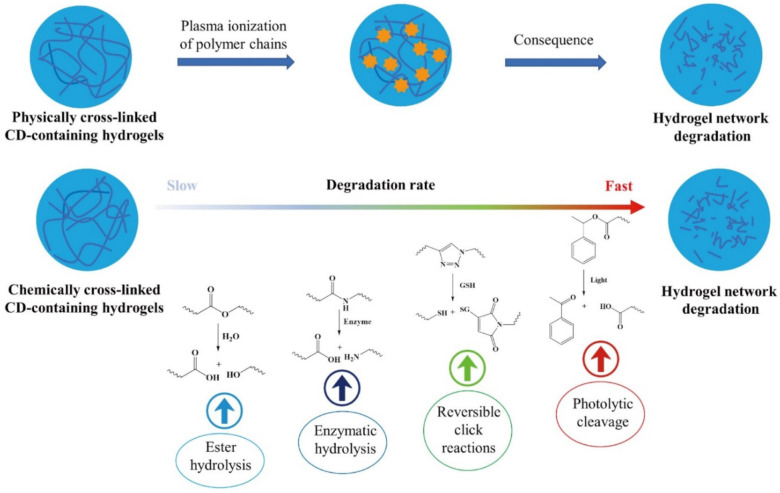
Simulated degradation behavior for CD-containing hydrogels.

The degradation process of hydrogel is one of the key indicators of its safety, and it is also the basis for studying in vivo decomposition of the hydrogel [113,114,115]. In vitro degradation behavior is studied through changes of the hydrogel under conditions such as simulated body fluids, different types of biological enzymes, and different acids and bases (Figure 2).

A previous study placed CD-based hydrogel loaded with gellan gum in a phosphate buffer solution (pH = 7.4), and the weight loss of the hydrogel was measured at 1, 7, 14, 21 days. Analysis showed that the weight loss rate of the hydrogel was low with the content of CD and that the weight loss rate of the modified hydrogel was higher compared with hydrogels without CD. This phenomenon can be attributed to the reduction of cross-linking sites in the modified hydrogel that lead to more unassociated chains in the gel network. In addition, hydrogels without CD had a slower degradation rate compared with those with CD, implying that the presence of CD in the matrix may increase the decomposition rate of the hydrogel [52]. Exposure of different hydrogels to the external environment, such as a phosphate buffer (pH = 7.4), showed that hydrogels with large cross-linking concentrations required longer hydrolysis time. Unfortunately, the higher cross-linking degree of the hydrogels caused more resistance to degradation in collagenase solution in PBS buffer. The cross-linking could limit the accessibility of enzymes to the cleavage sites of the hydrogels, and prevent the enzymes from penetrating the bulk of the material [116]. The degradation mechanism of these hydrogels mainly resulted from the breaking of cross-linking sites, ester bonds, hydrazone bonds, or steric hindrance through chemical hydrolysis. The polymer backbone was then hydrolyzed and broken into lower molecular weight and soluble fragments [117,118,119,120,121,122,123].

Degradation of hydrogel under pure water conditions was simulated, and the degradation rate of gallic acid-loaded hydrogel was explored to determine the environmental safety of bio-based hydrogels. Analysis showed that degradation of the hydrogel was relatively fast in the initial stages, however, it remained stable within 8–360 h. This degradation behavior can be attributed to the effect of water penetration in the bacterial cellulose hydrogel network which resulted in the good degradation behavior of hydrogel. Moreover, the aromatic ring (the presence of gallic acid and CD), slightly slows the degradation ability of the hydrogel. The aromatic ring may delay the degradation of the hydrogel [55]. 

The ability of hydrogels to degrade in the presence of organisms is important for drug release. A previous study evaluated the degradation of CD-based hydrogel using *Penicillium*, *Aspergillus niger*, and mushroom. Analysis showed that the gel changed to a liquid after six days of degradation. The degradation rate of the hydrogel increased gradually in *Penicillium*. In *Penicillium*, the degradation rate was 6.11% after three days, whereas after 21 days the degradation rate was 21.49%. In *Aspergillus niger*, the degradation efficiency of the hydrogel increases rapidly with time. The degradation rate was 15.9% at three days and 68.7% at 21 days. In addition, the degradation effect of mushrooms was higher compared with that of other strains. The experimentally prepared hydrogel was biodegradable and had a good degradation rate in mushrooms. Therefore, even if the hydrogel is disposed to the natural environment, the threat to the environment is minimal [124]. A different study used lysozyme to degrade the cyclic oligosaccharide backbone of a hydrogel by enzymatically hydrolyzing the glycosidic bonds on the hexasaccharide ring. The weight of the hydrogel decreased, indicating that the hydrogel was biodegradable. In addition, analysis of the infrared spectra of the degradation products (obtained on day 14 and day 28) showed that the C-O-C tensile peak intensity of the degradation products decreased after 14 days, whereas the C-O-C tensile peak disappeared after 28 days of degradation. At the same time, analysis of the day 14 and day 28 SEM images of the degraded hydrogel showed that the porous network morphology of the hydrogel was deformed and degraded, showing the degradability property of the hydrogel [125].

When preparing CD-loaded hydrogels, materials that are easily degraded in vivo should be selected as preparation materials. A poly[(R)-3-hydroxybutyrate] fragment was introduced into a hydrogel containing CD and analysis showed that the copolymer produced was biodegradable under physiological conditions. In addition, the hydrogel formulation was bioabsorbable after administration and was able to dissociate into its components [126]. However, materials used in some heat-sensitive hydrogels are not biodegradable [65]. A previous study explored a hydrogel that could be hydrolyzed by α-amylase after the introduction of CD. Moreover, the degradation rate increases when part of the poly-*N*-propylacrylamide molecular chain is replaced by maleic anhydride-β-CD (MAH-β-CD), mainly due to an increase in the hydrolysis of β-CD by α-amylase. Analysis of ^1^H NMR spectrum shows β-CD reacts with at least 2 maleic anhydride (MAH) molecules on average. Therefore, MAH-β-CD is a better cross-linking agent in the hydrogel network. At 37 °C, all hydrogels are in a semi-swelled state, and the speed at which enzymes enter the internal network of the hydrogel slows down [127].

When the degradation behavior of hydrogels is studied in an organism, the hydrogel is placed on the organism and then changes of the hydrogel at different times are observed [128]. Using mice as the model to verify the degradation of hydrogel, the degradation rate of the hydrogel was found to be faster under irradiation than without irradiation, indicating that the injectable hydrogel could be rapidly decomposed by photothermal treatment. Moreover, the fluorescence decay rate of the hydrogel was faster after irradiation than without irradiation. On the 4th day, the relative fluorescence signal intensity of the irradiated hydrogel group was about 60%, and the relative fluorescence signal intensity of the non-irradiated hydrogel group was close to 80%. On the 8th day, the relative fluorescence signal intensity of the non-irradiated hydrogel group was only about 40%, whereas the relative fluorescence signal intensity of the irradiated hydrogel group was about 20%. Therefore, laser irradiation can accelerate the decomposition of the hydrogel nanocomposite system. Light response assisted fluorescence imaging allows visualization of the disassembly of medical biomaterials. The near-infrared light-responsive supramolecular nanocomposite system is widely used to explore the fluorescent imaging tracking process of biomedical materials. The visualization of degradable supramolecular hydrogels on demand helps to eliminate carriers. The controllable non-invasive supramolecular nanocomposite is a promising biomedical material that allows instant stimulation and sustainable monitoring [129]. In another experiment on the degradation of hydrogels in mice during transdermal administration, Zhou et al. discovered that long-term biodegradation of hydrogels administered transdermally in mice may be caused by cross-linked hydrogel side chains. The existence of the cyclodextrin cavity made it difficult for the biological enzymes in the body to contact the hydrogel [96].

## 5. Conclusions and Perspectives

As a host molecule that can form host-guest inclusion complexes, CD has great application potential in the fields of medicine and materials science. Promisingly, the administration of CD-containing hydrogels can avoid the annoying side effects of traditional drug administration. In recent years, several studies have shown that hydrogel systems composed of CDs can effectively deliver drugs into target tissues. Especially, many physical and chemical methods have been successfully developed for preparing CD hydrogels.

Presently, the research on CD-containing hydrogel mainly focused on the field of biomedicine. In this review, different methods to design and prepare CD hydrogels are discussed. In addition, the potential applications of drug-loaded CD hydrogels are summarized. The data reviewed here show that introduction of CD improves the properties of the hydrogel and the drug release time of the hydrogels. Interestingly, some CD-containing hydrogels based on light and heat response have been successfully synthesized. This review broadens our understanding of the development trends in the application of CD-containing hydrogels, which lays the foundation for future clinical research. At the same time, looking forward to the future medical frontiers, we believe that an increasing number of emerging technologies will be introduced to guide the preparation of CD-containing hydrogels.

Future studies should design drug-loaded CD-containing hydrogels which can withstand factors in the external environment and internal environment in the body to guarantee excellent drug-release dynamics and drug-loading capacity. Moreover, it is of great importance to establish novel methods and tools for monitoring the metabolism of drugs in the body when drug-loaded CD-containing hydrogels enter the body. Meanwhile, attention should be paid to the safety issues associated with hydrogel materials inside the body. How the CD-containing hydrogels are degraded in the body, how they change, and whether they will interact with the body should be closely analyzed. Gratifyingly, more smart stimuli-responsive hydrogels based on CD for drug delivery will be designed and prepared, which can respond to different external and interior environmental stimuli to release drugs. In the future, we can actively take a page from cutting-edge technologies to prepare the CD-containing hydrogels for precision medicine that can accurately load appropriate doses of drugs. In addition, we should have a deep insight into the basic principles of drug loading and release of CD-containing hydrogels in the aspect of site-targeting, release conditions, dosing intervals for optimal treatment to different diseases. If the above-mentioned problems are fully solved, CD-containing hydrogels are expected to be more widely applied in clinical practice.

## Figures and Tables

**Table 1:** Comparison of cyclodextrin-containing hydrogels with other types of hydrogels.

Material for Forming Hydrogel	Cyclodextrin	Chitosan	Cellulose	Alginic Acid	Gum Arabic	Polyacryl Amide	Polyvinyl Alcohol
Source	Starch	Chitin	Plant	Alga	Acacia trees	Acrylonitrile	Vinyl acetate
Connection type	α-1, 4-glycosidic bond	β-1, 4-glycosidic bond	β-1, 4-glycosidic bond	1, 4-glycosidic bond	-	-	-
Techniques	Radical polymerization; Click reaction; Nucleophilic addition/substitution	Photo- polymerization; Thermal polymerization	Chemical crosslinking; Free-radical polymerization; Grafting; Freeze-thaw	Enzymatically crosslinking; Chemical crosslinking	Photo-induced radical polymerization	Radiation-induced	Freeze-thaw
Kinds of drug delivery	Hydrophobic drug	Small molecules; peptides; proteins	Small molecules; peptides; proteins	Traditional low-molecular-weight drugs and macromolecules	Small molecules; proteins	Small molecules; peptides; proteins	Small molecules; peptides; proteins
Clinic trial	Yes	Yes	Yes	Yes	No	Yes	Yes
Ref.	[27,28,29]	[30,31]	[32,33]	[34,35]	[36]	[37,38]	[39]

**Table 2:** The preparation methods of cyclodextrin-containing hydrogels.

Types	Matrix	Preparation Methods	Characteristic	Ref.
Physically cross-linked cyclodextrin-containing hydrogels	Chitosan	Casting method	Bilayer hydrogels	[48]
	Chitosan	Freeze-thaw cycling method	pH sensitivity	[49]
	Chitosan	Freezing method	Thermosensitive; Shortly gelation time (3 min or less)	[50]
	Chitosan/Poly(Vinyl Alcohol)	Dry at room temperature in vacuo	pH-specific release behavior	[51]
	Gellan gum	Gelation at room temperature	Biocompatible material	[52]
	Hydroxypropyl methylcellulose	Dispersion method	Benefit for skin	[53]
	Nanocellulose	30 min with autoclaving (121 ℃, 103 kPa)	Sustained release	[54]
	poly (vinyl alcohol)	Freezing drying method	Long-term release	[55]
	Soy soluble polysaccharide	Reduced pressure and stored in a desiccator	3D-nanocomposites, superabsorbent, malleable, bioadhesive	[56]
	Xanthan	Freezing drying method	Long-term release	[57]
Chemically cross-linked cyclodextrin-containing hydrogels	4-arm-Polyethylene glycol-Succinimidyl Glutarate	Nucleophilic substitution-based method	Improved the therapeutic effect	[58]
	Agarose gel	Nucleophilic substitution-based method	Low gelling temperature for controlled drug delivery	[59]
	Carboxymethyl cellulose	Free radical polymerization crosslinking-based method	pH-responsive behaviour	[60]
	Carboxymethyl cellulose	Nucleophilic substitution-based method	Biocompatible, capable of controlling the release for a long duration	[61]
	Chitosan	Nucleophilic substitution-based method	Local antibiotic release	[62]
	Nanocellulose	Nucleophilic substitution-based method	Cell compatibility, non-cytotoxicity	[63]
	Polyvinyl alcohol	Nucleophilic substitution-based method	Good strength, elasticity, WVP, and swelling ability	[64]
	Poly(*N*-isopropylacrylamide)	Free radical polymerization crosslinking-based method	Thermoresponsive	[65]
	Poly(2-hydroxyethyl methacrylate)	Nucleophilic substitution-based method	Sustained drug delivery	[66]
	Sodium hyaluronan	Nucleophilic substitution-based method	Controlled release	[67]

**Table 3:** Comparison of the properties of CD-containing hydrogels loaded with drugs [74,75,76,77,78,79].

Property	Physical Cross-Linked Hydrogel	Chemical Cross-Linked Hydrogel
Size of guest molecules	Small molecules (lipophilic)	Small molecules (lipophilic)
Drug loading strategies	Encapsulation	Encapsulation
Drug release speed	Can be controlled	Can be controlled
Drug release possible mechanisms	External stimulus; competition of external molecules	External stimulus; competition of external molecule
Duration times	Hours to days	Days to months
Drug delivery characteristic	High drug loading effectivity; low chance of drug deactivation	High drug loading effectivity; low chance of drug deactivation
Potential application	Drug delivery systems, injectable, wound dressings	Transdermal drug delivery, injectable, implantable, oral/ophthalmic drug carrier
Advantages	Non-toxic; cross-linking is reversible	Strong mechanical strength; the pore size can be adjusted; the variety of synthesis methods; difficult to degrade
Disadvantages	Low mechanical strength; difficult to adjust the pore size	Potentially toxic; no cross-linking is reversible

**Table 4:** Potential application of physically cross-linked CD-containing hydrogels.

No.	Drug	Formation Materials	Hydrogel State	Type of Cells	Summary	Potential Application	Ref.
1	Berberine hydrochloride	β-CD; Bacterial cellulose;	Nano- particle	*S. aureus*; *P. aeruginosa*; *E. coli*	The ultra-fine network of bacterial cellulose resulted in different release characteristics of berberine hydrochloride. The drug-loaded hydrogel had a good antibacterial effect as revealed by in vitro experiments.	Oral administration medicine	[54]
2	Chlorhexidine	β-CD; NaCl; NaHCO_3_; CaCl_2_	Contact lenses	*E. coli*	β-CD in eye drops significantly enhanced the delivery of chlorhexidine into the cornea.	Ocular delivery	[81]
3	Coumestrol	Hydroxypropyl-β-CD; methylcellulose	Not mentioned	Animals	Hydrogel has high efficacy in wound healing when compared to Dersani, with 50% wound healing achieved within a shorter period compared to this positive control.	Wound dressing materials	[53]
4	Curcumin	Hydroxypropyl-β-CD; silver nanoparticles; bacterial cellulose	Film	*P. aeruginosa*; *S. aureus*; *C. aureus*; Panc 1, U251, MSTO	The nano-silver particles loaded into the bacterial cellulose hydrogel showed high cytocompatibility and therapeutic effects against three common wound infection pathogens.	Wound dressing materials	[82]
5	Curcumin	β-CD; Polyvinyl alcohol	Film	Glioblastoma cell line C6; melanoma cell line B16F10; astrocyte cells	The hydrogel controlled the release of curcumin (48 h, 85% release). The polymer membrane had higher cytotoxicity than curcumin. The drug-loaded hydrogel showed prolonged cytotoxic effects (up to 96 h) at a lower concentration (50 μg/mL).	Local drug delivery system to treat cancer	[83]
6	Curcumin	2-hydroxypropyl-β-CD; sodium alginate; chitosan	Film	*E. coli*; *S. aureus*; NCTC clone 929 cells; NHDF cells	High concentration of crosslinking agent concentration improved the mechanical properties of the hydrogel and decreased the hygroscopicity, water swelling, and weight loss. In addition, hydrogel showed a slow-release effect (t > 50 h). Curcumin-loaded double-layer hydrogel effectively treated *E. coli* and *S. aureus*. The double-layer hydrogel was not toxic to NCTC clone 929 cells and normal human dermal fibroblasts.	Wound dressing materials	[48]
7	Gallic acid	Hydroxypropyl-β-CD; bacterial cellulose; poly (vinyl alcohol)	Not mentioned	Not mentioned	The swelling properties during encapsulation were inferior. The release profile of the complex was slower compared with gallic acid.	Pharmaceutical and cosmetic products	[55]
8	Honey bee propolis extract	β-CD; *κ*-Carrageenan	Not mentioned	*S. aureus*; *P. aeruginosa*; *Aspergillus* *Flavus*; *Candida albicans*	Higher active compound concentration ensures sustained in vitro release.	Wound dressing	[80]
9	Levofloxacin; methotrexate	Hydroxypropyl-β-CD; xanthan gum	Film	*E. coli*; *S. aureus*	The hydrogel loaded with the methotrexate showed a well-controlled release profile (t > 600 min). The hydrogel loaded with levofloxacin had a good antibacterial effect.	Drug delivery system	[57]
10	Red thyme oil	γ-CD; polyvinyl alcohol; chitosan; clinoptilolite	Film	L929 cells	Hydrogels with clinoptilolite contained characteristics such as compressed structure, improved mechanical properties, decreased swelling values, and reduced release rate of the drug. In addition, prepared hydrogels were low-toxic based on MTT assay.	Drug delivery systems and wound dressings	[84]
11	Thyme oil	Methyl-β-CD; hydroxypropyl-β-CD; γ-CD; chitosan; polyvinyl alcohol	Film	*E. coli*; *S. aureus*	The water vapor transmission rate of the hydrogel was appropriate for application in wound dressing. The swelling degree of hydrogel loaded with thyme oil varied with the pH. The hydrogels containing γ-CD had good antibacterial activity.	Wound dressings	[49,85]

**Table 5:** Potential application of chemically cross-linked CD-containing hydrogels.

No.	Drug	Formation Materials	Hydrogel State	Types of Cell	Summary	Potential Application	Ref.
1	5-Fluorouracil	β-CD; N-vinylcaprolactam; *N, N*′-methylene bisacrylamide	Nanogel	Human colon cancer cell lines (HCT 116); MRC-5 normal cells	The hydrogel had the best drug loading (659.7 mg/g) after controlling the feeding ratio. The drug release curve showed that the hydrogel could continue to release drugs for up to 30 h; especially in the intestinal juice with pH = 7.4, the 5-fluorouracil drug molecules contained therein were not completely released; and the maximum release rate was 68%.	Implantable hydrogels	[93]
2	Coumarin	β-CD; alginate; calcium homopoly-L-guluronate	Supramolecular hydrogel	RAW 264.7 cells; T. cruzi cells	The lowest release of substituted amidocoumarins from the hydrogels occurred at pH = 1.2 whereas the maximum release (34%) was observed at pH 8.0.	Biomedicine	[94]
3	Curcumin	β-CD; epiclon	Nanosponge	Non-tumorigenic human breast; invasive mouse breast cell lines (4T1)	The high degree of cross-linking led to the formation of mesoporous having high specific surface area and high loading capacity. Nanosponge showed no toxicity against MCF 10A and 4T1 cells as normal and cancerous cells, respectively.	Cancer therapy	[95]
4	Curcumin	Carboxymethyl-β-CD; gelatin; methacrylic anhydride	Microneedle arrays	B16F10 melanoma cell	The inclusion complex of curcumin maintained 90% of the initial concentration. Besides, the hydrogel could enhance the drug loading and adjust release. In vivo study showed that hydrogel had good biocompatibility and degradability.	Transdermal drug delivery	[96]
5	Dexamethasone	β-CD; low-acyl gellan gum; EDC	Injectable hydrogel	NIH/3T3 mouse embryo fibroblast	After drug loading, the gel-forming temperature of the modified hydrogel was reduced and the mechanical properties are improved. Hydrogel had a high affinity and release rate for drugs. In vivo studies had shown that the drug-loaded hydrogel improved the anti-inflammatory response.	Tissue engineering and regenerative medicine	[52]
6	Dexamethasone	β-CD; sodium hyaluronate	Delivery hydrogel	3T3 cells	The novel hydrogels significantly improved the therapeutic effect of dexamethasone in burn wound healing.	Wound healing	[58]
7	Dexibuprofen	β-CD; acrylic acid; methylene bisacrylamide	Nanosponges	Not mentioned	The solubility of ibuprofen in the hydrogel was increased 6.3 times. In vitro release experiments demonstrated that the drug release rate of β-CD nanosponges reached 89% within 30 min under the condition of pH = 6.8.	Oral administration of lipophilic drugs	[97]
8	Diclofenac sodium	β-CD; sodium hyaluronan; EDC;	Contact lens materials	*S. aureus*; 3T3 fibroblasts	The hydrogel not only reduced the adsorption of tearing proteins due to electrostatic mutual repulsion but also improved encapsulation capacity and sustainable release of diclofenac (t > 72 h). In vitro cell viability analysis displayed that all hydrogels were non-toxic to 3T3 mouse fibroblasts.	Ophthalmic diseases	[66]
9	Doxorubicin	β-CD; 2-ethyl-2-oxazoline; aminopropyltriethoxy silane; FeCl_2_.4H_2_O; FeCl_3_.6H_2_O	Magnetic nanohydrogel	MCF7 cells	The magnetic nanohydrogel had a good drug loading rate (74%) and encapsulation rate (81%). Under acidic conditions (pH = 5.3), adding a small amount of GSH (10 mM) increased the release value (89.21%). The magnetic nanohydrogel had good cell compatibility even at high concentrations (10 mg/mL).	Implantable hydrogels	[98]
10	Doxorubicin	β-CD; agarose	Injectable hydrogel	Human embryonic kidney 239 cells; HeLa cells	The hydrogel was able to easily and uniformly load a drug at 30 °C. The drug-loaded hydrogel maintained the drug’s anti-cancer activity. In addition, the hydrogels did not exhibit toxicity toward the HEK-293 and HeLa cells.	Injectable hydrogel	[59]
11	Doxorubicin	β-CD; hyaluronic acid; bis(4-nitrophenyl) carbonate	Injectable hydrogel	Human colorectal cancer cells HCT-116	Rheological tests showed that this hydrogel could be easily prepared and used on a schedule compatible with normal operating room procedures. In vitro experiments showed that the unique physical and chemical properties of the hydrogel ensured the sustained release of anticancer drugs (t > 32 d) and prevented the growth of colorectal cancer micelles under 3D culture conditions.	Device for localized chemotherapy of solid tumors	[99]
12	Doxorubicin; curcumin	β-CD; multiwalled carbon nanotubes; maleic anhydride; folic acid; hexamethylene diisocyanate	Nanocarrier	Not mentioned	This injectable hydrogel exhibited pH/thermo response and exerted a deleterious effect on the tumor. A sustained release of the two drugs was observed over a period of 30 h. The release rate of doxorubicin reached 90% under tumor microenvironmental conditions, and the release rate of curcumin reached 85% under high temperature and physiological pH conditions.	Injectable nanocarriers	[100]
13	Doxorubicin	β-CD; tetronic; adamantane	Injectable shear-thinning hydrogels	HeLa cell	The hydrogels showed shear-thinning behaviors, rapid recovery properties, pH-responsive properties, and long-term release of the hydrophobic drug.	Embolic material	[101]
14	Insulin	Carboxymethyl β-CD; carboxymethyl chitosan	Microparticles	Caco-2 cells	The insulin was loaded into the hydrogel, and the results of the drug release experiment found that the insulin was successfully retained in the stomach environment and slowly released after passing through the intestine. In vitro studies had shown that the hydrogel particles exhibited non-cytotoxicity and were mainly transported in the Caco-2 cell monolayer through paracellular pathways.	Oral drug delivery	[102]
15	Vitamin E	β-CD; soy soluble polysaccharides; galacturonic acid	Core-shell bionanomaterials hydrogel	Not mentioned	The hydrogel exhibited significant swelling adsorption and sustained release (t > 230 h) for the release of vitamin E in vitro. The encapsulation efficiency and drug loadings were 79.10% and 16.04%, respectively. In addition, after oral administration of the vitamin E-loaded hydrogel in rats, the vitamin E level in the plasma continued to increase within 12 h.	Oral drug carrier	[56]

## Data Availability

Not applicable.

## Abbreviation

FTIR	Fourier transform infrared spectroscopy
NMR	nuclear magnetic resonance
SEM	scanning electron microscope
XRD	X-Ray diffraction
*S. aureus*	*Staphylococcus aureus*
*P. aeruginosa*	*Pseudomonas aeruginosa*
*E. coli*	*Escherichia coli*
*C. aureus*	*Staphylococcus aureus enterotoxin C*
Panc 1	Human pancreatic cancer cell
U251	Human brain glioma U251 cell line
MSTO	human mesothelioma
NHD	Fskin fibroblast cells
L929 cells	L929 mouse fibroblast cells
